# Methane Emissions and Microbial Communities as Influenced by Dual Cropping of *Azolla* along with Early Rice

**DOI:** 10.1038/srep40635

**Published:** 2017-01-17

**Authors:** Jingna Liu, Heshui Xu, Ying Jiang, Kai Zhang, Yuegao Hu, Zhaohai Zeng

**Affiliations:** 1 College of Agriculture and Biotechnology, China Agricultural University, Beijing, China; 2 Plant & Soil Science Section, Department of Plant and Environmental Science, Faculty of Science, University of Copenhagen, Frederiksberg, Denmark

## Abstract

*Azolla caroliniana* Willd. is widely used as a green manure accompanying rice, but its ecological importance remains unclear, except for its ability to fix nitrogen in association with cyanobacteria. To investigate the impacts of *Azolla* cultivation on methane emissions and environmental variables in paddy fields, we performed this study on the plain of Dongting Lake, China, in 2014. The results showed that the dual cropping of *Azolla* significantly suppressed the methane emissions from paddies, likely due to the increase in redox potential in the root region and dissolved oxygen concentration at the soil-water interface. Furthermore, the floodwater pH decreased in association with *Azolla* cultivation, which is also a factor significantly correlated with the decrease in methane emissions. An increase in methanotrophic bacteria population (*pmoA* gene copies) and a reduction in methanogenic archaea (16S rRNA gene copies) were observed in association with *Azolla* growth. During rice cultivation period, dual cropping of *Azolla* also intensified increasing trend of 1/Simpson of methanogens and significantly decreased species richness (Chao 1) and species diversity (1/Simpson, 1/D) of methanotrophs. These results clearly demonstrate the suppression of CH_4_ emissions by culturing *Azolla* and show the environmental and microbial responses in paddy soil under *Azolla* cultivation.

Rice fields are generally considered a major source of atmospheric methane (CH_4_)^1^. The global CH_4_ emission rate from paddies was estimated to be 20–40 Tg per year^2^,^3^, which accounts for approximately 11% of the total methane emissions^4^. The global warming potential (GWP) of CH_4_ is 25 times greater than that of CO_2_ on a mass basis and 100-year time horizon^5^. Therefore, management strategies to mitigate the CH_4_ emissions from paddy fields have attracted intensive studies.

*Azolla* is a heterosporous pteridophyte with a wide distribution in temperate and tropical aquatic ecosystems, such as swamps, ditches and lakes^6^,^7^. Due to its symbiosis with *Anabaena azollae*, a N_2_-fixing cyanobacterium, *Azolla* has been employed as a companion crop for rice in Asia for centuries, and it is believed to be a sustainable natural source of N^8^. Additionally, *Azolla* can retard NH_3_ volatilization by lowering the floodwater pH when urea is applied and serves as a temporary nitrogen reserve material^9^. Previously, it was reported that *Azolla* simultaneously cultured with rice could significantly lower CH_4_ emissions in studies conducted in eastern India^10^ and southern China^11^, but opposite results were reported in the studies conducted in northeastern China^6^,^12^. These studies demonstrated that the effects of *Azolla* on methane emissions may vary among different environments, and more information regarding the environmental response to *Azolla* cultivation is required.

Methane production, transport and oxidation in paddy soils are strongly influenced by environmental variables, including temperature, soil redox potential (Eh), rice variety, pH, fertilizer type and other factors^13^,^14^,^15^,^16^. The high dissolved O_2_ in floodwater can mitigate methane emissions in two ways: directly stimulating methane oxidation at the soil-water interface^17^ and indirectly inhibiting methane production and promoting methane oxidation in flooded paddy soils by increasing soil Eh. Conversely, low levels of dissolved O_2_ contribute to increased methane emissions^18^. The variation in dissolved O_2_ in floodwater under the presence of *Azolla* is also controversial: it increased in some studies^10^,^11^ and decreased in others^6^,^12^. Nevertheless, these previous studies only focused on the environmental variables affected by dual cropping of *Azolla*, but the methanogenic archaea and methanotrophic bacteria in paddy soils influenced by cultivating *Azolla* have not yet been investigated. The net balance between methane produced by methanogens and oxidized by methanotrophs determines the CH_4_ emissions from paddy fields^18^,^19^,^20^. To identify the key *Azolla*-induced parameters for the mitigation or simulation of CH_4_ emissions, a microbiological study investigating the dynamics of methanogenic and methanotrophic communities affected by dual cropping of *Azolla* in rice fields is necessary.

The community diversity of methanogenic archaea and methanotrophic bacteria in paddy soils has been extensively investigated with PCR-DGGE (Polymerase Chain Reaction-Denaturing Gradient Gel Electrophoresis) and T-RELP (Terminal Restriction Fragment Length Polymorphism) analysis methods^21^,^22^,^23^. With the potential to obtain a complete coverage of the microbial community^24^, high-throughput sequencing may improve the characterization of the microbial community^25^. In the present study, the Illumina MiSeq sequencing approach was employed to investigate the community structure of methanogenic archaea and methanotrophic bacteria in conjunction with the physiochemical analysis of water and soil in a flooded paddy with dual cropping of *Azolla*. In addition, methane emission rates were measured using the static chamber method, while the quantities of 16S rRNA gene for methanogenic archaea and *pmoA* gene for methanotrophic bacteria were estimated using quantitative PCR.

## Results

### Seasonal dynamics of methane flux

**Figure 1:**
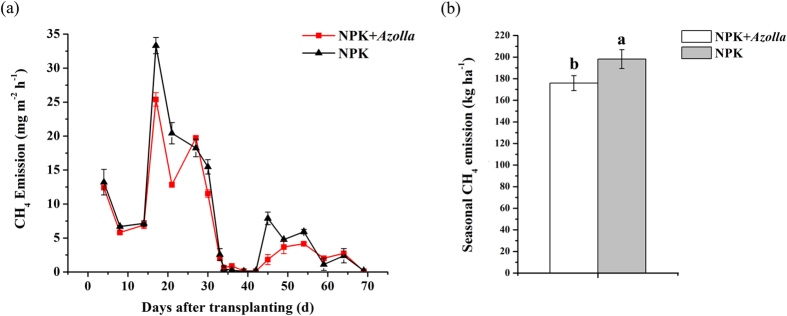
The seasonal dynamics of the CH_4_ emission rate (**a**) and cumulative CH_4_ emissions (**b**) from paddy soil influenced by the cultivation of *Azolla*. NPK, rice cropping without dual cropping of *Azolla* under recommended fertilization; NPK + *Azolla*, rice cropping with dual cropping of *Azolla* under recommended fertilization. The CH_4_ emission rate is the mean of the values measured within each treatment (n = 3). Vertical bars denote the standard deviation of the means. Different letters above the error bars denote a significant difference between the two treatments at the same sampling time (n = 3, *p* < 0.05, *T*-test).

The CH_4_ flux trends were similar in the NPK and NPK + *Azolla* treatments (Fig. 1a). A significant CH_4_ flux was initiated on day 15 after rice transplantation and peaked on day 17, with 33.3 mg m^−2^ h^−1^ and 25.4 mg m^−2^ h^−1^ for NPK and NPK + *Azolla* treatments, respectively. Thereafter, the methane flux decreased sharply to a minimum, near zero, during the midseason drainage (days 34–42), recovered immediately when the paddy soil was flooded again, and finally decreased gradually as the maturity stage approached. Moreover, the CH_4_ flux was considerably lower in NPK + *Azolla* than in NPK. Additionally, consistently lower cumulative CH_4_ emissions were observed in the treatment with dual cropping of *Azolla* (175.9 kg ha^−1^), significantly reducing the CH_4_ emissions by 11.2% relative to those of NPK (Fig. 1b).

### Paddy soil and floodwater parameters

In general, slightly acidified bulk soil pH values (0.1–0.4 units, ranging from 6.3 to 7.0), significantly decreased floodwater pH values (*p* < 0.05, 0.3–1.0 units, ranging from 6.0 to 7.0), notably increased DO (dissolved oxygen) concentrations (*p* < 0.05, 20.8–58.3%), and higher root region Eh (3.0–13.1%) were observed in NPK + *Azolla* compared with NPK during the rice growth period (from day 10 to day 67) (Table 1).

**Figure 2:**
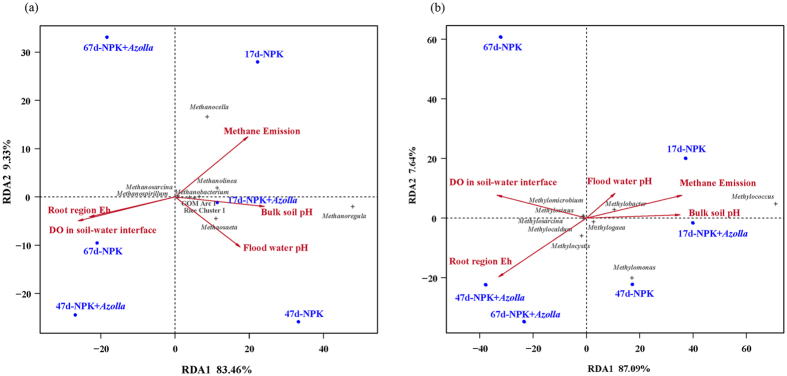
Redundancy analysis (RDA) displaying the relationship among environmental parameters (red), paddy soil samples (blue) and relative abundances (gray) of methanogenic archaeal (**a**) and methanotrophic bacterial (**b**) communities classified at the genus level. Only the genera known to be methanogens (**a**) and methanotrophs (**b**) are shown on the RDA plot. NPK, rice cropping without dual cropping of *Azolla* under recommended fertilization; NPK + *Azolla*, rice cropping with dual cropping of *Azolla* under recommended fertilization.

The correlation analysis indicated an extremely significant positive correlation between the CH_4_ emission rate and the pH values of the bulk soil and floodwater (*p* < 0.01, R = 0.614/0.466, n = 16, respectively) and significant negative correlations between the CH_4_ flux and the DO concentration at the soil-water interface or rhizosphere soil Eh (*p* < 0.05, R = −0.433 and −0.531, n = 16 and 14, respectively). These relationships between environmental parameters and methane emissions have also been demonstrated in an RDA analysis (Fig. 2).

### Abundance of methanogens and methanotrophs in paddy soil

**Figure 3:**
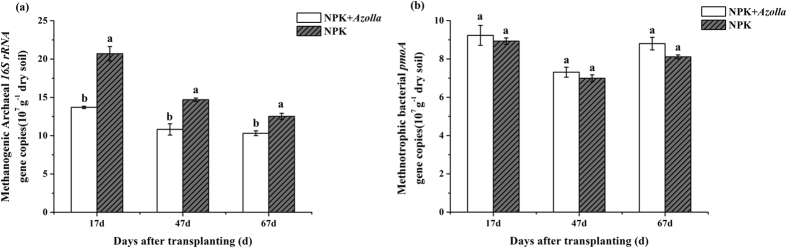
Relative abundances of methanogenic archaeal *16S rRNA* genes (**a**) and methanotrophic bacterial *pmoA* genes (**b**) based on quantitative PCR in the paddy soil of the two treatments during the rice cultivation period (days 17, 47 and 67 after early rice transplantation). Vertical bars indicate the standard deviation of the means (n = 3). Different letters above error bars denote a significant difference between the two treatments at the same sampling time (n = 3, *p* < 0.05, *T*-test). NPK, rice cropping without dual cropping of *Azolla* under recommended fertilization; NPK + *Azolla*, rice cropping with dual cropping of *Azolla* under recommended fertilization.

The qPCR results (Fig. 3) showed that the methanogenic 16S rRNA gene copies were significantly decreased (*p* < 0.05, 33.8%, 26.4% and 17.6%) (Fig. 3a) and that the methanotrophic *pmoA* genes were slightly increased (3.4%, 4.6% and 8.5%) (Fig. 3b) on the days 17, 47, and 67 (the rice growth period), respectively, in NPK + *Azolla* compared with in the NPK.

### Changes in methanogenic archaeal communities

Dual cropping of *Azolla* along with rice had a moderate effect on the alpha diversity of the methanogenic community in paddy soils during the rice cultivation period (Table 2). In total, 135, 354 high-quality reads (average length: 296.72 bp) were obtained after the optimization process, and a total of 65 OTUs (at the 97% similarity cutoff) across all the samples were confirmed to belong to methanogens. The coverage values close to 1 among all the samples (0.9996–0.9999) reflected the high depth of sequencing. Sampling time and the presence of *Azolla* had no significant effect on methanogenic species richness (Chao 1) (Table 3). Methanogenic species diversity (1/Simpson, 1/D) remarkably increased with rice cultivation period but showed no significant difference with or without *Azolla* cultivation. However, interaction was observed between sampling time and *Azolla* management. Treatments with *Azolla* cultivation intensified increasing trend of 1/Simpson.

**Figure 4:**
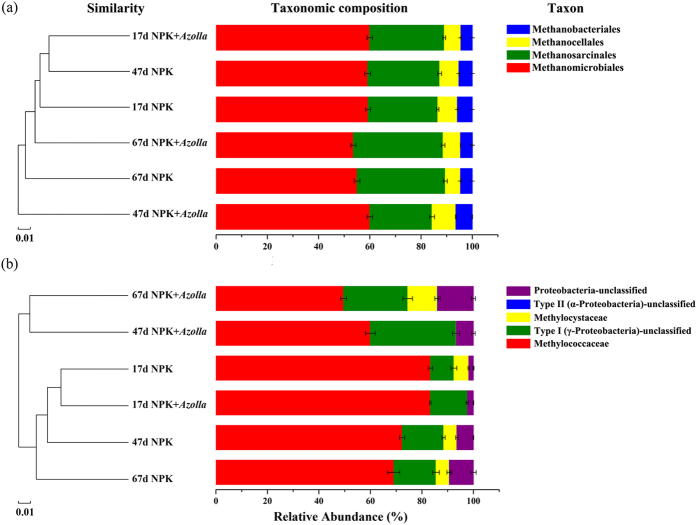
Cluster analysis (left) combined with taxonomic composition barplot (right) of methanogenic archaea (**a**) and methanotrophic bacteria (**b**). NPK, rice cropping without dual cropping of *Azolla* under recommended fertilization; NPK + *Azolla*, rice cropping with dual cropping of *Azolla* under recommended fertilization.

**Figure 5:**
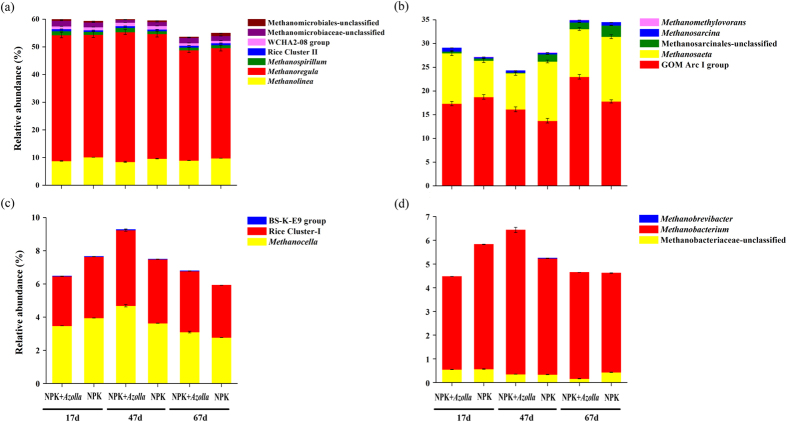
Variations in the communities of Methanomicrobiales (**a**), Methanosarcinales (**b**), Methanocellales (**c**) and Methanobacteriales (**d**) detected in the paddy soil during the rice cultivation period. Methanogenic archaeal 16S rRNA gene sequencing reads were classified at the genus level using the RDP Classifier (http://rdp.cme.msu.edu/) against the silva (SSU115) 16S rRNA database; only the genera known to be methanogens are shown. NPK, rice cropping without dual cropping of *Azolla* under recommended fertilization; NPK + *Azolla*, rice cropping with dual cropping of *Azolla* under recommended fertilization.

Hierarchical clustering analysis demonstrated that methanogenic archaeal communities were highly similar among all the samples. ANOSIM analysis was used to testify if methanogenic archaeal samples clustered according to the presence of *Azolla* or cultivation period (Table 4). Samples were grouped according to with or without *Azolla* and cultivation period (17d, 47d and 67d), ANOSIM analysis indicated that cluster according to the presence of *Azolla* and cultivation period were both significant (*p* < 0.05) based on genus level. The methanogenic community consisted of Methanomicrobiales (53.6% ± 0.6–60.0% ± 0.6%), Methanosarcinales (24.3% ± 0.6–35.0% ± 0.4%), Methanocellales (5.9% ± 0.005–9.3% ± 0.08%) and Methanobacteriales (4.5% ± 0.005–6.4% ± 0.06%) (Fig. 4a). Dual cropping of *Azolla* primarily affected Methanosarcinales and had little influence on the other three. Representing the second dominant methanogenic population, Methanosarcinales mainly consisted of *Methanosaeta* (7.6% ± 0.3–13.6% ± 0.2%) and the GOM Arc I group (13.7% ± 0.3–23.0% ± 0.3%), which were sensitive to the *Azolla* cultivation (Fig. 5b). Compared with NPK, the relative abundance of *Methanosaeta* increased on day 17 (+3.0%, *p* < 0.05) and decreased on days 47 (−4.9%, *p* < 0.05) and 67 (−3.7%, *p* < 0.05). In contrast, the relative abundance of the GOM Arc I group decreased on day 17 (−1.4%, *p* < 0.05) and increased on days 47 (+3.0%, *p* < 0.05) and 67 (+2.4%, *p* < 0.05). The most predominant methanogenic population, Methanomicrobiales, mainly consisted of *Methanolinea* (8.4% ± 0.1–10.1% ± 0.007%) and *Methanoregula* (39.9% ± 0.6–46.9% ± 0.5%) (Fig. 5a). During the rice growth period, dual cropping of *Azolla* significantly (*p* < 0.05) increased the relative abundance of *Methanoregula* by 1.2% and 2.1% on days 17 and 47, respectively.

Furthermore, the RDA plot (Fig. 2a) demonstrated that the pH, including the bulk soil pH and floodwater pH, had possible correlations with the relative abundance of most methanogens, including *Methanosaeta*, the GOM Arc I group, *Methanosarcina, Methanospirillum*, and Rice Cluster I.

**Figure 6:**
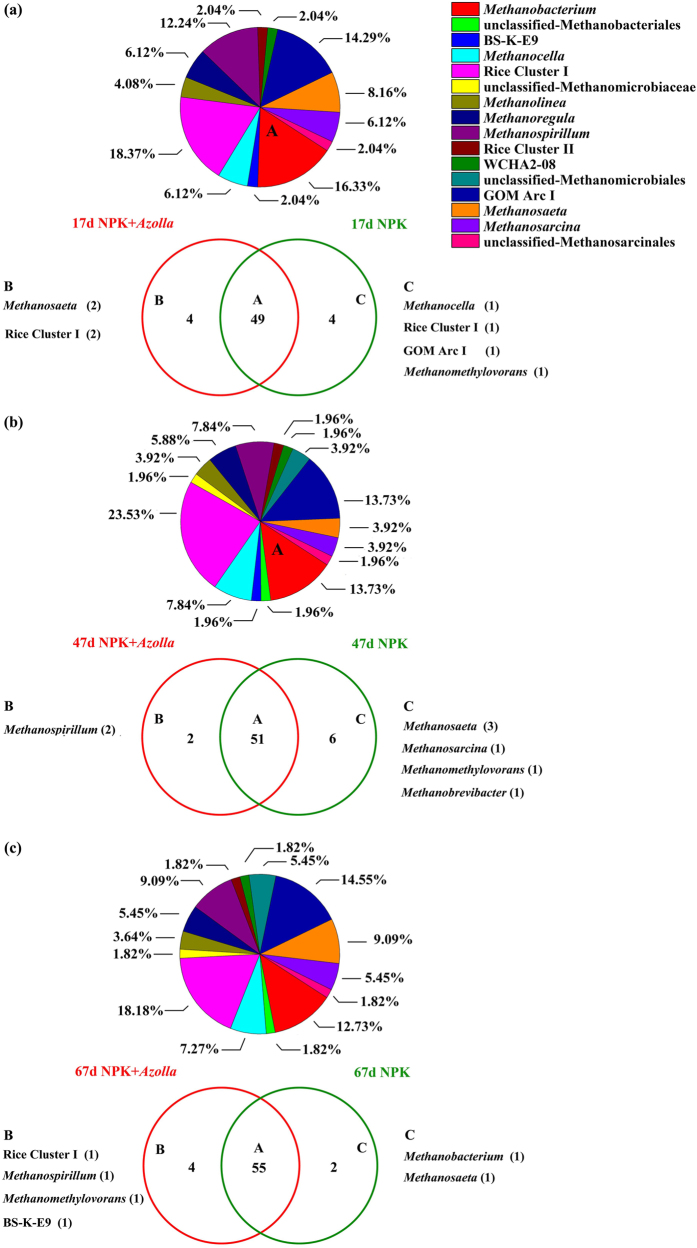
Venn diagrams and pie charts showing the distribution of methanogenic archaeal 16S rRNA OTUs in the two treatments across three sampling times: day 17 (**a**), day 47 (**b**) and day 67 (**c**) after rice transplantation. The numbers within the diagrams indicate the number of OTUs shared between two treatments (A) or unique to the given treatment (B,C) (unique OTUs were identified as unique that were found in all three replicates of one treatment but not in any of the triplicates of the other treatment). The pie charts denote the taxonomic identity and distribution of shared OTUs (A). Unique OTUs are listed beside the Venn diagrams (B,C). NPK, rice cropping without dual cropping of *Azolla* under recommended fertilization; NPK + *Azolla*, rice cropping with dual cropping of *Azolla* under recommended fertilization.

Analysis of the shared and unique OTUs revealed that dual cropping of *Azolla* barely affected the methanogenic archaeal OTU distribution (Fig. 6). There were in a total of 57, 59 and 61 OTUs from the two treatments (NPK and NPK + *Azolla*) on days 17, 47, and 67, respectively, and shared OTUs represented 86.0%, 87.9% and 90.2%, respectively. Four unique OTUs (unique OTUs were identified as unique that were found in all three replicates of one treatment but not in any of the triplicates of the other treatment.) on day 17, 2 unique OTUs on day 47, and 4 unique OTUs on day 67 were detected in NPK + *Azolla*.

### Changes in methanotrophic communities

Dual cropping of *Azolla* along with rice had a significant impact on the alpha diversity of the methanotrophic community in paddy soils during the rice cultivation period (Table 2). In total, 137, 145 high-quality reads (average length 299.98 bp) were obtained after the optimization process, and a total of 198 OTUs across all samples were defined belonging to methanotrophs. Coverage values in all samples approached 1 (0.9993–0.9999), reflecting the high depth of sequencing. Species richness (Chao 1) was significantly decreased by by 35.2% and 13.4% on days 17 and 47, respectively, under NPK + *Azolla* treatment. The species diversity (1/Simpson, 1/D) also decreased on days 17, 47, and 67 compared with NPK. Two factorial analyses of variance (ANOVA) revealed remarkable changes in the methanotrophic bacterial diversity (Chao 1, 1/D) under dual cropping of *Azolla* (Table 3). The hierarchical clustering analysis indicated that the methanotrophic bacterial communities were affected by the growth of *Azolla* because the communities of these bacteria were similar during the initial days (day 17) in both treatments but were clearly different in the samples from the later days (days 47 and 67) from the two treatments (Fig. 4b). ANOSIM analysis was carried out to test if methanotrophic bacterial samples clustered according to the presence of *Azolla* or cultivation period (Table 4). Samples were grouped according to with or without *Azolla* and cultivation period (17d, 47d and 67d), ANOSIM analysis indicated that cluster according to the presence of *Azolla* and cultivation period were both significant (*p* < 0.05) based on OTU and genus level.

**Figure 7:**
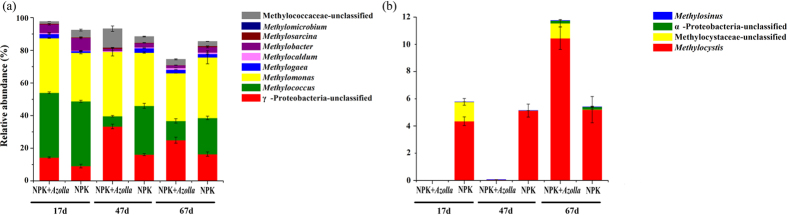
Variations in the communities of Type I methanotrophs (**a**) and Type II methanotrophs (**b**) in the paddy soil during the rice cultivation period. Methanotrophic bacterial *pmoA* gene sequencing reads were classified at the genus level using the RDP Classifier (http://rdp.cme.msu.edu/) against the FGR functional gene database; only the genera known as methanotrophs were shown. NPK, rice cropping without dual cropping of *Azolla* under recommended fertilization; NPK + *Azolla*, rice cropping with dual cropping of *Azolla* under recommended fertilization.

In terms of community composition, the methanotrophic community consisted of Type I methanotrophs, Type II methanotrophs and unclassified methanotrophs (Fig. 4b). Type I methanotrophs were the overwhelmingly predominant populations, with much higher relative abundances (74.4% ± 1.9–97.6% ± 0.13%) than those of Type II methanotrophs (<0.01–11.8% ± 1.1%) during the rice cultivation period. Type I methanotrophs mainly consisted of *Methylomonas* (29.2% ± 0.002–39.4% ± 2.5%), *Methylococcus* (6.3% ± 0.4–39.7% ± 0.6%), *Methylobacter* (1.5% ± 0.003–7.8% ± 0.6%) and unclassified Methylococcaceae (1.3% ± 0.002–11.4% ± 1.7%) (Fig. 7a). Type II methanotrophs mainly consisted of *Methylocystis* (<0.01–10.5% ± 0.8%) (Fig. 7b). Compared with NPK, the NPK + *Azolla* treatment had a much lower Type II methanotroph relative abundance (<0.01%, 0.06% ± 0.006%) and a correspondingly higher Type I methanotroph relative abundance (97.6% ± 0.1%, 93.2% ± 0.7%) on days 17 and 47. In contrast, on day 67, a higher Type II methanotroph relative abundance (11.8% ± 1.1%) and a correspondingly lower Type I methanotroph relative abundance (74.4% ± 1.9%) were observed in the NPK + *Azolla* treatment. For the Type I methane oxidizer, cultivating *Azolla* significantly (*p* < 0.05) increased the relative abundance of *Methylomonas* (+3.8% and +6.8% on days 17 and 47, respectively) and dramatically (*p* < 0.05) decreased the relative abundance of *Methylococcus* (−23.5% and −10.5% on days 47 and 67, respectively). Moreover, after being strongly inhibited, Type II methanotrophs, mainly *Methylocystis* (10.5% ± 0.8%), increased on day 67 under dual cropping of *Azolla*.

The RDA plot (Fig. 2b) demonstrates that the relative abundances of *Methylobacter* and *Methylogaea* were possibly correlated with methane emissions and pH values, including bulk soil pH and floodwater pH values. In contrast, *Methylomicrobium, Methylosinus, Methylosarcina* and *Methylocaldum* were probably correlated with root region Eh and dissolved oxygen concentration at the soil-water interface.

**Figure 8:**
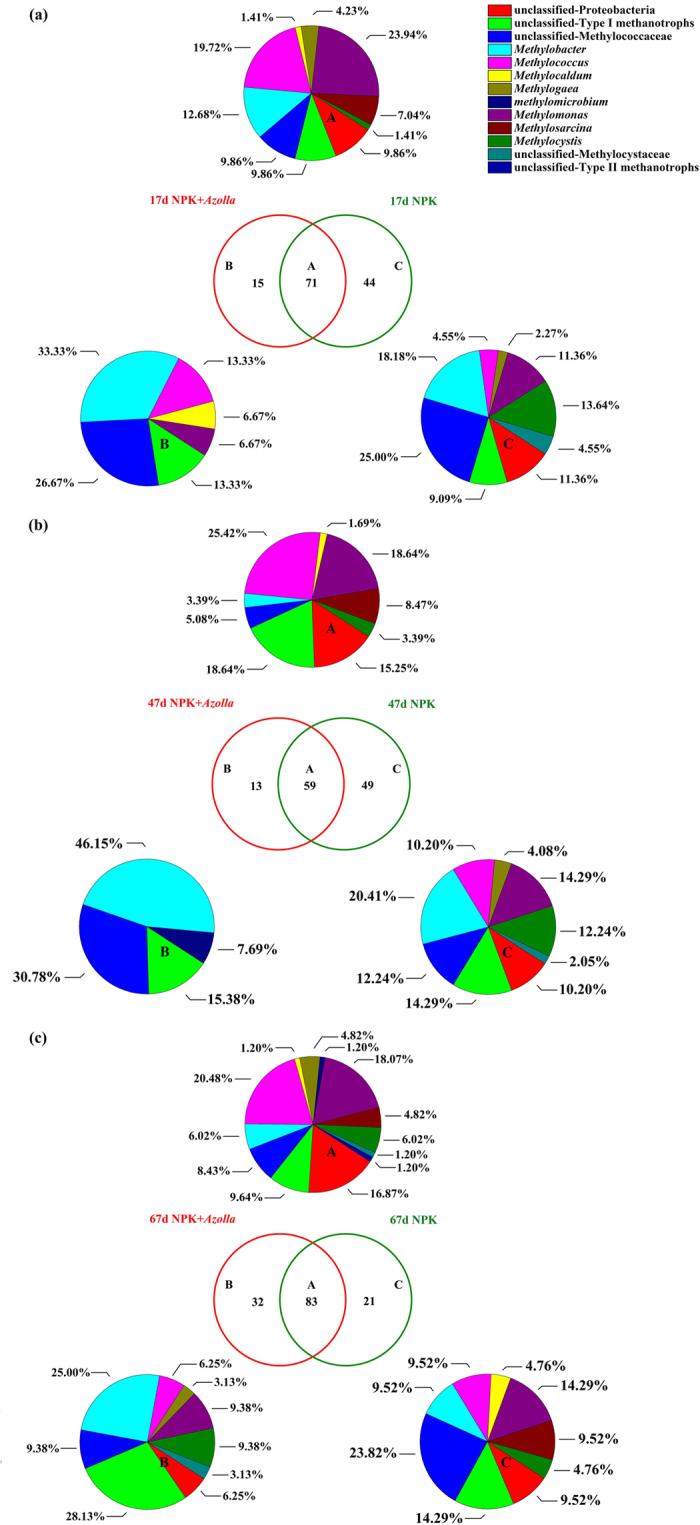
Venn diagrams and pie charts showing the distribution of methanotrophic *pmoA* OTUs in the two treatments across three sampling times, day 17 (**a**), day 47 (**b**) and day 67 (**c**) after rice transplantation. The numbers within the Venn diagrams indicate the number of OTUs shared between two treatments (A) or unique to the given treatment (B,C) (unique OTUs were identified as unique that were found in all three replicates of one treatment but not in any of the triplicates of the other treatment). The pie charts denote the taxonomic identity and distribution of shared (A) or unique (B and C) OTUs. NPK, rice cropping without dual cropping of *Azolla* under recommended fertilization; NPK + *Azolla*, rice cropping with dual cropping of *Azolla* under recommended fertilization.

Analysis of the shared and unique OTUs revealed that dual cropping of *Azolla* also had an apparent effect on methanotrophic bacterial OTU distribution (Fig. 8). Totals of 130, 121 and 136 OTUs were obtained from the two treatments (NPK, NPK + *Azolla*), and shared OTUs represented 54.6%, 48.8% and 61.0% on days 17, 47, and 67, respectively. NPK + *Azolla* had 15 unique OTUs (unique OTUs were identified as unique that were found in all three replicates of one treatment but not in any of the triplicates of the other treatment.) on day 17, 13 unique OTUs on day 47, and 32 unique OTUs on day 67. In general, the unique OTUs in NPK + *Azolla* mainly belonged to unclassified Gamma-Proteobacteria, unclassified Methylococcaceae and *Methylobacter*.

## Discussion

The influence of *Azolla* as a dual crop in rice cultivation on communities of methanogenic archaea and methanotrophic bacteria was investigated in present study using Illumina MiSeq sequencing in conjunction with measurements of methane emission rates and paddy environmental factors across the entire rice growth period. Based upon the analyses of soil and floodwater properties, the reduced methane emissions in the present study can be linked to the modifications of environmental factors and the microbial communities.

The pH value is an environmental parameter that is correlated with CH_4_ production in rice paddies and also acts as one of key determinants and predictors of soil archaeal and bacterial communities^26^,^27^. The optimal pH for methanogens is 6.9–7.1, and lower pH values are usually associated with low methanogenic activity and reduced methane emissions from paddy soil^28^,^29^. In the present study, the significant acidification of the floodwater and slight acidification of the paddy soil due to the culturing of *Azolla* (Table 1) may partially explain the reduced methane emissions. The decrease in the pH of the floodwater caused by cultivating *Azolla* has been reported in some previous studies^30^,^31^. The pH decrease may be related to the fact that the floating *Azolla* absorbs most available solar radiation and consequently limits the photosynthesis of algae in the water^30^,^32^, which subsequently lowers the consumption of CO_2_ in the floodwater and thus the pH. Furthermore, the *Azolla*-*Anabaena* complex, which is independent of the CO_2_ in the floodwater, continues to photosynthesize and fix nitrogen without elevating the floodwater pH^33^. Additionally, the *Azolla*-*Anabaena* complex might release CO_2_ derived from respiration into the floodwater. Therefore, the increasing CO_2_ concentration in the floodwater might directly and indirectly contribute to the reduction of floodwater pH and bulk soil pH, respectively.

The low Eh in flooded soil is vital to the normal functioning of methanogens due to their anaerobic characteristics. Soils with low Eh values are usually correlated with high methane emissions^10^,^18^. In addition, the oxygen is crucial to methanotrophic bacteria as an activator for methane oxidation but also very important as an electron acceptor^18^. In general, following flooding, the soil Eh decreases immediately^18^, but the decline in the Eh is adversely affected by O_2_ diffusion in the surface soil layer and percolation of oxygenated floodwater in flooded paddy soil^17^,^34^. The dissolved oxygen in floodwater was higher in the treatment with dual cropping of *Azolla*, creating a more oxidized state at the soil-water interface and in the flooded paddy soil (higher Eh), as revealed in this study. This effect promoted methane oxidation and inhibited methane production. Compared with NPK, the decrease in the methane emission rate and cumulative emissions of NPK + *Azolla* after rice transplantation is likely to be attributable to the depression of floodwater and bulk soil pH and the promotion of DO and root region Eh. An RDA plot (Fig. 2) and correlation analysis also confirmed the above relationships: pH (floodwater and bulk soil) had a significant positive correlation with methane emissions, while DO and Eh in the root region had a significant negative correlation with methane emissions.

The significant decrease in the methanogenic 16S rRNA gene copies revealed by qPCR in the NPK + *Azolla* treatment was also consistent with the reduced methane emissions and was possibly caused by the decrease in bulk soil pH and the increase in paddy soil Eh (Table 1), as estimated by Conrad^18^. The slight increase in methanotrophic *pmoA* gene copies observed in the NPK + *Azolla* treatment might be attributed to the more oxidized state (Table 1) and the fact that the *Azolla-Anabaena* symbiosis can provide fixed nitrogen to other bacteria, including methanotrophs^10^,^35^.

Regarding the methanogenic archaeal communities, the predominance of Methanomicrobiales, the stable structural compositions, and the stable relative abundances of different methanogenic groups during the entire rice growth period in both the treatments were similar to the previous results obtained under different management regimes^21^,^25^,^36^. Therefore, the members of Methanomicrobiales may possibly play a critical role in methane production in paddy soil regardless of the environmental changes. The genus *Methanoregula*, composed of hydrogenotrophic and acidophilic methanogens utilizing H_2_/CO_2_ as an energy substrate for methane production^18^,^37^, was predominant throughout the rice cultivation period, suggesting that H_2_/CO_2_ may be a crucial source of carbon and energy for methanogenesis. Moreover, the slight increase in *Methanoregula* in NPK + *Azolla* (Fig. 5a) might have been favored by the relatively low pH value in the NPK-*Azolla* treatment (Table 1). This finding is consistent with the RDA analysis that suggests that bulk soil pH is correlated with the relative abundance of *Methanoregula* (Fig. 2a). As the second predominant methanogenic group, the relative abundance of the order Methanosarcinales, especially the genus *Methanosaeta* and the GOM Arc I group, were apparently influenced by dual cropping of *Azolla* (Fig. 5b), indicating that *Methanosaeta* and the GOM Arc I group may be more sensitive to the environmental variations caused by dual cropping of *Azolla* than other methanogens. According to the RDA plot (Fig. 2a), the most highly correlated environmental variation was the decreased pH. In addition, as a genus of effective acetoclastic methanogens that can stay active at very low acetate concentrations (<100 μM)^18^,^38^, *Methanosaeta* was far more abundant than *Methanosarcina* in the present study (Fig. 7b), suggesting that the concentration of acetate may be very low. The orders Methanocellales and Methanobacteriales had low but constant relative abundances throughout the rice growth period (Fig. 5c and d).

For the methanotrophic bacteria, the considerable shift in the relative abundance of Type I and the constant relative abundance of Type II during the rice cultivation period (Fig. 7) were consistent with the results of previous reports^18^,^25^. Better adaptability and resistance to adverse conditions, such as desiccation and heat, may partially explain the stability of the Type II methanotrophs^39^. The genus *Methylococcus* was particularly predominant on day 17, when the methane emission rate was high, and its relative abundance decreased dramatically on days 47 and 67, when the methane emission rates were low (Fig. 7a), especially in the NPK + *Azolla* treatment. Therefore, the members of *Methylococcus* may be responsible for a great proportion of the methane oxidation and may require higher methane concentrations for growth. Additionally, the RDA plot demonstrates that the methane emission rates were correlated with the relative abundance of *Methylococcus* (Fig. 2b). The genus *Methylocystis* barely existed in NPK + *Azolla* on days 17 and 47, when *Azolla* was growing well, and then exhibited a striking increase on day 67, when the drainage was performed (Fig. 7b), suggesting that *Methylocystis* may require more oxidative environments. The RDA plot (Fig. 2b) also indicates that this genus was correlated with the increased Eh in the root region and greater dissolved oxygen concentration in the floodwater.

In addition to microbial communities of methanogens and methanotrophs, cultivating *Azolla* along with rice may affect methane transport from the soil to the atmosphere. Besides diffusion into the atmosphere via the aerenchyma of the rice plant, ebullition through the formation of gas bubbles is an important process for methane transport and contributes 2.5–15% of the total CH_4_ emissions^18^,^40^. Free-floating *Azolla* cover may serve as physical barrier which could block the ebullition when it reaches high enough density and large percentage cover^41^,^42^.

The significantly lower (11.9%) methane emissions associated with dual cropping of *Azolla* in the present study (Fig. 1) are consistent with the previous reports of Bharati *et al*.^10^ and Ma *et al*.^11^ but in contrast to those of Chen *et al*.^12^ and Ying *et al*.^6^. The contrary results may be due to different climate conditions resulting in different dual cropping duration and growth status of *Azolla*. Both Chen *et al*.^12^ and Ying *et al*.^6^ performed field trials in northeastern China, Chenyang, where the temperature during the rice growing season is 9–24 °C^6^. Generally, the optimum temperature for *Azolla* growth is between 18 °C and 28 °C^8^. Therefore, southern China, where the temperature during rice growing season is 22–29 °C (present study, Fig. S1), may be a more suitable area for the growth of *Azolla* than northeastern China. Ying *et al*.^6^ also indicated that *Azolla* may not prevent CH_4_ transport from floodwater into the atmosphere, but the coverage density of *Azolla* was 0.31 kg m^−2^ in their pot trial and the dual cropping effect was tested for only 24 h. In the present study, the coverage density of *Azolla* reached 0.75 ± 0.18 kg m^−2^ above the floodwater 19 d after rice transplantation, and the dual cropping effect on CH_4_ emissions was evaluated over 69 d. Better growth of *Azolla* may serve as physical barrier to block the methane ebullition^41^,^42^ and also favor the decrease of pH in floodwater and the increase of soil Eh and DO in floodwater which benefits the methane mitigation.

The present study indicated that dual cropping of *Azolla* could significantly decrease the methane emissions from paddy fields, and the possible explanations might be the external changes in the environmental factors, environment-induced variations in microbial community structure and the inhibition of CH_4_ transport via ebullition. Considerably decreased bulk soil pH and floodwater pH influenced the relative abundance of most methanogens (e.g., *Methanoregula, Methanosaeta*, the GOM Arc I group, etc.) and some methanotrophs (*Methylococcus, Methylobacter* and *Methylogaea*), and may have led to the decreased methanogenic archaeal 16S rRNA gene copies. Markedly increased root region Eh and dissolved oxygen concentration of the soil-water interface were correlated with most methanotrophs (e.g., *Methylomonas, Methylocaldum, Methylocystis*, etc.), and may have contributed to the increased methanotrophic bacterial *pmoA* gene copies. Furthermore, it is noteworthy that dual cropping of *Azolla* strongly inhibits the growth of *Methylocystis*.

Additionally, further study and application of dual cropping *Azolla* along with rice is meaningful for minimizing CH_4_ emissions from flooded paddy soil worldwide. According to the FAO statistics from 2012 (http://faostat.fao.org), China, India, Indonesia, Bangladesh and Vietnam were the top 5 rice-producing countries in the world, contributing 71.9% of global rice production, and generated 15.2 Tg CH_4_ emissions from paddy fields in 2012 (61% of the global CH_4_ emissions from paddies in 2012). In addition, most of the rice cultivation areas (tropical, subtropical and warm-temperature regions) in these five countries are suitable habitats for *Azolla*. The dual cropping of *Azolla* and rice may act as a practical mitigation option and may result in a methane emissions reduction of approximately 1.7 Tg per year from flooded paddies (according to the 2012 data from the FAO, http://faostat.fao.org).

## Materials and Methods

### Rice field experiments

A field experiment was conducted in an experimental station at the Institute of Soil and Fertilizer in Dongting Lake Plain, Academy of Agricultural Science of Hunan Province, China (29°52′N, 112°55′E), in 2014. The climate in this region is semi-tropical, humid and monsoonal, with an average frost-free period of 260–310 d and 5273 degrees of accumulative temperature (≥10 °C) per year. The mean annual temperature is 16–18 °C, and the average annual rainfall is 1200–1700 mm. The monthly mean air temperature and precipitation during January 2014 to December 2014 are shown in Fig. S1. The soil was classified as purple calcareous clayey paddy soil, and its properties were as follows: soil organic matter, 49.2 g kg^−1^; pH, 7.1; total nitrogen, 3.11 g kg^−1^; available nitrogen, 273.0 mg kg^−1^; available phosphorous, 16.4 mg kg^−1^; and available potassium, 69.0 mg kg^−1^.

A single-factor randomized block design with three replications was applied to investigate the effect of *Azolla* cultivation on methane emissions from the paddies. Two treatments were established: 1) rice cropping without dual cropping of *Azolla* under recommended fertilization (NPK) and 2) rice cropping with dual cropping of *Azolla* under recommended fertilization (NPK + *Azolla*). The field was divided into plots (4 m × 6 m) separated by 0.5 m ridges. *Azolla caroliniana* Willd. was allowed to grow after being inoculated into NPK + *Azolla* plots at 1 Mg ha^−1^ 7 d after rice transplantation (the coverage density of *Azolla* was 0.76 kg m^−2^ when it reached full coverage above the floodwater, 12 d after inoculation). Rice seedlings (cv. Luliangyou 996, early rice, 30 d; 2 × 10^5^ seedlings per hectare) were transplanted on May 2 and harvested on July 16. A recommended basal dose of 85 kg P_2_O_5_ ha^−1^ and 100 kg K_2_O ha^−1^, provided by calcium superphosphate and potassium chloride, respectively, was applied to each treatment when the rice seedlings were transplanted. Urea was applied at rates of 70 and 30 kg ha^−1^ N on the rice transplantation day and peak tillering stage, respectively. During the rice growth period, normal field management strategies were followed, including weeding, irrigation (maintaining a floodwater depth of 10 cm), mid-season aeration (from day 34 to day 42 after rice transplantation) and drainage in the post-maturation stage (day 66 after rice transplantation).

### Measurement of methane emission rates

The static chamber method^1^,^10^,^25^ was employed to measure the methane emission rates. Six basins (2 treatments × 3 replications) were embedded at 5 cm depth in the paddy soil with four growing rice plants in each after the transplantation of rice seedlings. When gas sampling (every 3–7 d during 9:00–11:00 in the morning), a plexiglass chamber (45 × 45 × 100 cm^3^) was installed onto the basin. During drainage, the basin was filled with water, forming an airtight seal. Moreover, a small fan was installed in the top of the chamber to mix headspace gases and keep the temperature constant during sampling. The air temperature inside chamber was measured using a manual thermocouple thermometer (JM624, Tianjin Instrument Co. Ltd., Tianjin, China). Gas samples (50 mL each) were extracted from the chamber with injectors at 5, 25 and 45 min after closure. Air samples was immediately transferred to 0.5 L evacuated sample bags (Dalian Hede Technologies Ltd. Dalian, China) and taken instantly to lab for CH4 measuring. CH_4_ concentrations were determined using a gas chromatograph (Shimadzu, GC-2010, Japan) equipped with a flame ionization detector (200 °C). The CH_4_ emission rate was calculated according to the following equation:


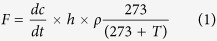


here, *F* is the CH_4_ flux (mg m^−2^ h^−1^), dc/dt is the slope of the curve of the gas concentration versus time, *h* is the headspace height (m) of the chamber, ρ is the gas density (kg m^−3^) at standard state, and *T* is the air temperature (°C) inside the chamber.

### Analysis of soil and floodwater properties

After rice transplantation, paddy soil and floodwater properties were measured every 5–15 d. For soil pH measurements, triplicate bulk soil was sampled in paddy fields for each treatment (10 g, 1–10 cm depth) and extracted for 1 h at room temperature with distilled water (water:soil, 2.5:1), and the pH value of the filtered extracts was measured using a portable high-precision instrument for the multi-parameter analysis of water quality (HANNA, HI9829-04, Italy). The floodwater pH and dissolved oxygen at the soil-water interface were determined *in situ* (from six randomly picked points in paddy fields for each treatment) with the same apparatus, HI9829-04. The Eh of the root region (approximately 10 cm depth) was directly measured *in situ* (from six randomly picked points near rice plants for each treatment) using the depolarization method with an automatic ORP tester (CN61M/FJA-6, China).

### Soil sampling

Soil sampling was conducted on day 17 (tillering stage), day 47 (heading stage), and day 67 (mature stage) after rice transplantation. At each sampling time, for one replication, 6 plants per treatment were uprooted and the loosely bound soil was shaken off. Then, the soil closest to the root was gently removed and carefully collected and mixed together to form a composite sample (be subdivided into three equivalent splits for further use). All soil samples (2 treatments × 3 replications (composite sample) × 3 times) were immediately frozen in liquid nitrogen and stored at −20 °C after being taken to the laboratory.

### DNA extraction and quantitative PCR assay

Two weeks after collecting the soil samples, the total soil DNA was extracted from each of the 18 soil samples using 0.5 g soil as the input into a FastDNA Spin Kit (MP Biomedicals, USA) according to the manufacturer’s instructions. The purity and concentration of the extracted soil DNA were determined with a NanoDrop ND-1000 UV-Vis Spectrophotometer (Thermo Fisher Scientific, USA). The quantified DNA extracts were stored at −20 °C until further use.

For quantitative PCR assay, the specific primer sets 1106 F/1378 R (5′-TTW AGT CAG GCA ACG AGC-3′ and 5′-TGT GCA AGG AGC AGG GAC-3′) and A189F/mb661R (5′-GGN GAC TGG GAC TTC TGG-3′ and 5′-CCG GMG CAA CGT CYT TACC-3′) were used for methanogenic archaeal 16S rRNA genes and methanotrophic bacterial *pmoA* genes, respectively^43^,^44^. The quantitative PCR reactions were performed on an ABI 7500 Fast Quantitative PCR system (Applied Biosystems, USA) using SYBR green as the detection system. The quantitative PCR program was 95 °C for 3 min, followed by 40 cycles at 95 °C for 30 s, 57 °C for 20 s and 72 °C for 30 s for methanogenic archaea and 95 °C for 3 min, followed by 40 cycles at 95 °C for 10 s, 55 °C for 30 s, and 72 °C for 30 s for methanotrophic bacteria.

The 25 μL reaction mixture was prepared using 12.5 μL of SYBR Premix Ex Taq II (TaKaRa, Japan), 1 μL of each primer (10 μM), 1 μL of template DNA, and 9.5 μL of sterile distilled water. All DNA samples from each treatment were analyzed in duplicate.

To generate standard curves, the positive clones KM015274 and KJ095325 from the environmental DNA sample were used for the methanotrophic bacterial *pmoA* gene and methanogenic archaeal 16S rRNA gene, respectively. Plasmids were extracted using a QIAprep Spin Miniprep Kit (OMEGA, USA), and DNA concentrations were determined with a NanoDrop ND-1000 UV-Vis Spectrophotometer (Thermo Fisher Scientific, USA). Tenfold serial dilutions ranging from 5.12 × 10^2^ to 5.12 × 10^8^ copies μL^−1^ and 3.72 × 10^2^ to 3.72 × 10^8^ copies μL^−1^ were used to construct standard curves for methanotrophic bacteria and methanogenic archaea, respectively. The qPCR amplification efficiencies of 99.5% (*R*^2^ = 99.8%) and 97.6% (*R*^2^ = 99.7%) were obtained for methanogenic archaea and methanotrophic bacteria, respectively. The copy numbers for the *pmoA* gene and methanogenic archaeal 16S rRNA gene were calculated according to the standard curve within the linear range. Fluorescence data were measured when the temperature was raised from 65 °C to 95 °C to confirm the PCR amplification specificity. CFX Manager™ software (version 1.6) was employed to perform thermal cycling, fluorescent data collection, and data analysis according to the manufacturer’s instructions. Three blank controls (no-DNA template) were run for each quantitative PCR assay. The extraction of DNA and determination of gene abundance were ensured with cautions to meet the requirement of MIQE guidelines^45^.

### PCR amplification and Illumina MiSeq sequencing

The PCR amplifications were performed on a Thermocycler ABI 9700 (Applied Biosystems, USA). The 16S ribosomal RNA gene of the methanogenic archaea and *pmoA* gene of the methanotrophic bacteria were amplified by PCR (95 °C for 5 min, followed by 30 cycles at 94 °C for 45 s, 57 °C for 45 s, and 72 °C for 90 s and a final extension at 72 °C for 10 min; 95 °C for 5 min, followed by 30 cycles at 95 °C for 60 s, 54 °C for 60 s, and 72 °C for 60 s and a final extension at 72 °C for 10 min) using the primers 1106 F/1378 R with a barcode at the 5′-end of 1106 F and *pmoA*f325/*pmoA*r643 (5′-barcode-TGG GGY TGG ACC TAY TTCC-3′ and 5′-CCG GCR CRA CGT CCT TACC-3′)^46^, respectively, where the barcode is an eight-base sequence unique to each sample. The PCR reactions were performed in triplicate using 20 μL mixtures containing 4 μL of 5× FastPfu Buffer, 2 μL of 2.5 mM dNTPs, 0.8 μL of each primer (5 μM), 0.4 μL of FastPfu Polymerase, and 10 ng of template DNA.

Amplicons were extracted from 2% (w/v) agarose gels and purified using the AxyPrep DNA Gel Extraction Kit (Axygen Biosciences, USA) according to the manufacturer’s instructions and quantified using QuantiFluor™-ST (Promega, USA). Purified amplicons were pooled in equimolar and paired-end sequenced (2 × 300 bp) on an Illumina MiSeq platform according to the standard protocols.

### Processing of Illumina MiSeq sequencing data

Raw fastq files were demultiplexed and quality filtered using QIIME (version 1.17) with the following criteria: (i) the reads with approximately 300 bp were truncated at any site receiving an average quality score <20 over a 10-bp sliding window, and the truncated reads that were shorter than 50 bp were excluded from further study; (ii) exact barcode matching, 2 nucleotide mismatch in primer matching, and reads containing ambiguous characters were removed; and (iii) only sequences that overlapped longer than 10 bp were assembled according to their overlap sequence. Reads that could not be assembled were discarded^47^.

Operational taxonomic units (OTUs) were clustered with 97% similarity cutoff using UPARSE (version 7.1 http://drive5.com/uparse/), and chimeric sequences were identified and removed using UCHIME. The phylogenetic affiliation of each 16S rRNA gene sequence and each *pmoA* gene sequence was determined with RDP Classifier (http://rdp.cme.msu.edu/) against the silva (SSU115) 16S rRNA database and FGR functional gene database, respectively, using a confidence threshold of 70%^48^,^49^.

The Chao 1 estimator of community richness, Simpson index of community diversity and Coverage indicating sequencing depth were chosen to evaluate community alpha diversity using the mothur program, version v.1.30.1 (http://www.mothur.org/wiki/Schloss_SOP#Alpha_diversity)^50^. Hierarchical clustering analysis (Jiang *et al*., 2013) was conducted based on UPGMA (unweighted pair group method with arithmetic mean) using QIIME (Bray-Curtis) for the calculation of beta diversity distance matrix. Venn diagrams^51^ and community composition histograms^52^ were generated using the ‘Vennerable’ package and ‘graphics’ package, respectively, in R, version 3.2.1^53^.

### Nucleotide sequence accession number

The MiSeq sequencing data of the 16S rRNA and *pmoA* genes are publicly available in the NCBI Short Read Archive (SRA) under accession no. SRP076506.

### Statistical analysis

Statistical analyses of the methane emission rate and abundance data were conducted using the LSD (least significant difference) test at the 0.05 probability level using IBM SPSS Statistics 20. According to the detrended correspondence analysis (DCA) of species-sample (OTUs with 97% similarity cutoff), the length of the gradient in the first axis indicated that redundancy analysis (RDA) could be used to investigate the correlations among environmental parameters, soil samples and methanogenic archaeal/methanotrophic bacterial communities. Therefore, in the R program environment (http://cran.r-project.org)^25^, RDA was conducted using the package ‘VEGAN’, and its statistical significance level was detected via the Permutest function^24^,^54^. Pearson correlation coefficients and *p* values between the environmental parameters and methane emission rates were calculated using IBM SPSS Statistics 20. Analysis of similarity (ANOSIM) tests using PRIMERv7 were carried out to determine whether there were assemblage differences between groups of samples specified according to dual cropping of *Azolla* or cultivation period. Based on OTU level (relative abundances of sequences from OTUs) and genus level (relative abundances of sequences from different genera), 999 permutation (higher than possible permutation) of the test for each ANOSIM analysis were conducted by using a resemblance matrix of Bray-Curtis dissimilarity as determined in mothur (after pre-transformation, log(X + 1)).

## Additional Information

**How to cite this article:** Liu, J. *et al*. Methane Emissions and Microbial Communities as Influenced by Dual Cropping of *Azolla* along with Early Rice. *Sci. Rep.* **7**, 40635; doi: 10.1038/srep40635 (2017).

**Publisher's note:** Springer Nature remains neutral with regard to jurisdictional claims in published maps and institutional affiliations.

## Supplementary Material

Supplemental Information

## Figures and Tables

**Table 1:** Environmental variations in the rice field under the influence of *Azolla* from day 10 to day 61 after early rice transplantation.

Treatment^a^	Days after rice transplanting								
	10	17	24	33	40	47	54	61	Mean
Paddy bulk soil pH value									
NPK + *Azolla*	7.00 ± 0.09a^b^	6.90 ± 0.07a	6.80 ± 0.19a	6.00 ± 0.09a	6.00 ± 0.11a	6.60 ± 0.23a	6.50 ± 0.15a	6.33 ± 0.22a	6.5
NPK	7.20 ± 0.19a	7.00 ± 0.09a	6.90 ± 0.07a	6.40 ± 0.20a	6.00 ± 0.10a	6.80 ± 0.06a	6.70 ± 0.18a	6.40 ± 0.20a	6.7
Floodwater pH value									
NPK + *Azolla*	6.48 ± 0.08b	6.42 ± 0.06b	7.05 ± 0.19b	6.62 ± 0.11b	6.73 ± 0.12b	6.82 ± 0.24b	6.63 ± 0.15a	6.33 ± 0.22b	6.6
NPK	7.12 ± 0.19a	7.21 ± 0.10a	8.00 ± 0.09a	7.52 ± 0.23a	7.21 ± 0.12a	7.32 ± 0.07a	6.89 ± 0.19a	6.82 ± 0.22a	7.3
DO at the soil-water interface									
NPK + *Azolla*	2.17 ± 0.21a	1.82 ± 0.2a	1.98 ± 0.21a	2.34 ± 0.13a	—	2.01 ± 0.14a	2.17 ± 0.14a	2.30 ± 0.16b	2.11
NPK	1.88 ± 0.16b	1.15 ± 0.05b	1.46 ± 0.08b	1.75 ± 0.20b	—	1.65 ± 0.08b	1.52 ± 0.05b	2.64 ± 0.12a	1.72
Eh in the root region of rice plants									
NPK + *Azolla*	−56.1 ± 3.2a	−135.6 ± 6.1a	−142.3 ± 2.8a	−176.3 ± 11.1a	−106.0 ± 6.8a	−115.8 ± 3.5a	−158.7 ± 7.6a	−113.3 ± 3.4a	−125.5
NPK	−53.0 ± 5.0a	−148.7 ± 3.2b	−155.8 ± 2.8b	−181.8 ± 7.3a	−110.7 ± 5.1a	−133.3 ± 14.5b	−171.8 ± 7.6b	−129.4 ± 3.7b	−135.6

^a^Each bulk soil pH value is the mean of 3 replications and the rest of environmental variables (floodwater pH, DO and Eh) values were the mean of 6 replications. NPK, rice cropping without dual cropping of *Azolla* under recommended fertilization; NPK + *Azolla*, Rice cropping with dual cropping of *Azolla* under recommended fertilization.

^b^In a column, values with different letters are significantly different (*p* < *0.05*) by one way Anova test (comparison between two treatments in same environmental factor measurement).

**Table 2:** Alpha-diversity of methanogenic archaeal and methanotrophic bacterial community during rice cultivation period (day 17, day 47 and day 67 after early rice transplantation).

		Reads^a^	0.97^b^		
Treatment			OTU	Chao	1/Simpson
Methanogenic archaea					
17d	NPK + *Azolla*	18397	53	57 ± 4	6.61 ± 0.19
	NPK	25814	53	54 ± 3	6.74 ± 0.19
47d	NPK + *Azolla*	20642	53	55 ± 6	6.69 ± 0.24
	NPK	26205	57	59 ± 3	6.81 ± 0.18
67d	NPK + *Azolla*	24242	59	61 ± 3	7.57 ± 0.24
	NPK	20054	57	60 ± 4	6.87 ± 0.19
Methanotrophic bacteria					
17d	NPK + *Azolla*	23911	86	90 ± 2	6.89 ± 0.19
	NPK	22116	115	142 ± 7	8.02 ± 0.27
47d	NPK + *Azolla*	23958	72	94 ± 5	11.7 ± 0.29
	NPK	23139	108	112 ± 10	12.7 ± 0.36
67d	NPK + *Azolla*	22100	115	119 ± 6	22.7 ± 0.67
	NPK	21921	104	116 ± 7	24.2 ± 1.05

^a^Sequence reads were the sum values of three replicates for each treatment.

^b^Alpha-diversity indices (Chao, community richness; Simpson, community diversity) of methanogenic archaeal and methanotrophic bacterial community in each sample were calculated using ‘mothur’. OTUs, Operational Taxonomic Units, were clustered with 97% similarity cutoff using UPARSE. OTU numbers in the column were the union OTU numbers of three replicates for each treatment.

**Table 3:** Results of the two factorial analyses of variance (ANOVA) for effect of rice cultivation period, *Azolla* and their two-way interactions on Chao and 1/Simpson of methanogenic archaeal and methanotrophic bacterial community.

Main factors and interactions	Methanogenic archaea								Methanotrophic bacteria							
	Chao				1/Simpson				Chao				1/Simpson			
	df^a^	*MS*^b^	*F*	*p*	df	*MS*	*F*	*p*	df	*MS*	*F*	*p*	df	*MS*	*F*	*p*
Time	2	39.5	2.37	ns^c^	2	0.52	12.59	<0.01	2	390.72	9.22	<0.01	2	405.69	1299.9	<0.01
*Azolla*	1	0	0	ns	1	0.1	2.42	ns	1	2222.22	52.42	<0.01	1	6.21	19.89	<0.01
Time × *Azolla*	2	19.5	1.17	ns	2	0.34	8.3	<0.01	2	1157.72	27.31	<0.01	2	0.09	0.29	ns
Residual	12	16.67			12	0.04			12	42.39			12	0.31		
Total	18				18				18				18			

^a^df: degree of freedom.

^b^MS: Mean square.

^c^ns: no significant (*p* > 0.05).

**Table 4:** Correlations between methanogenic archaeal and methanotrophic bacterial community similarity and the presence of *Azolla* or cultivation period of early-rice.

Domain			based on OTU level			based on Genus level		
	Grouping		ANOSIM R statistic	ANOSIM P-value	Significant?	ANOSIM R statistic	ANOSIM P-value	Significant?
Methanogens	*Azolla* (with or without *Azolla*)		0.125	0.072	No	0.278	0.013	Yes
	Cultivation period	17d × 47d	0.222	0.048	Yes	0.296	0.037	Yes
		17d × 67d	0.502	0.002	Yes	1	0.002	Yes
		47d × 67d	0.7	0.002	Yes	0.4	0.015	Yes
Methanotroph	*Azolla* (with or without *Azolla*)		0.352	0.003	Yes	0.187	0.047	Yes
	Cultivation period	17d × 47d	0.7	0.002	Yes	0.4	0.006	Yes
		17d × 67d	0.7	0.002	Yes	0.967	0.002	Yes
		47d × 67d	0.1	0.21	No	0.328	0.024	Yes

A 999 permutations of the test for each ANOSIM analysis (higher than possible permutations). A test is considered significant if *P* < 0.05.
