# Supplementary material for: Methane Emissions and Microbial Communities as Influenced by Dual Cropping of Azolla along with Early Rice
Source: Sci Rep. 2017 Jan 17;7:40635. doi: 10.1038/srep40635 (PMC5240575; doi:10.1038/srep40635)
Supplement: Supplemental Information [file srep40635-s1.doc]

**Methane Emissions and Microbial Communities as Influenced by Dual Cropping of *Azolla* along with Early Rice**

Jingna Liu^1,2,+^, Heshui Xu^1,+^, Ying Jiang^1^, Kai Zhang^1^, Yuegao Hu^1^, Zhaohai Zeng^1*^

^1^ College of Agriculture and Biotechnology, China Agricultural University, Beijing, China

^2^ Plant & Soil Science Section, Department of Plant and Environmental Science, Faculty of Science, University of Copenhagen, Frederiksberg, Denmark

^*^ Corresponding author, [zengzhaohai@cau.edu.cn](mailto:zengzhaohai@cau.edu.cn)

^+^ These authors contributed equally to this work


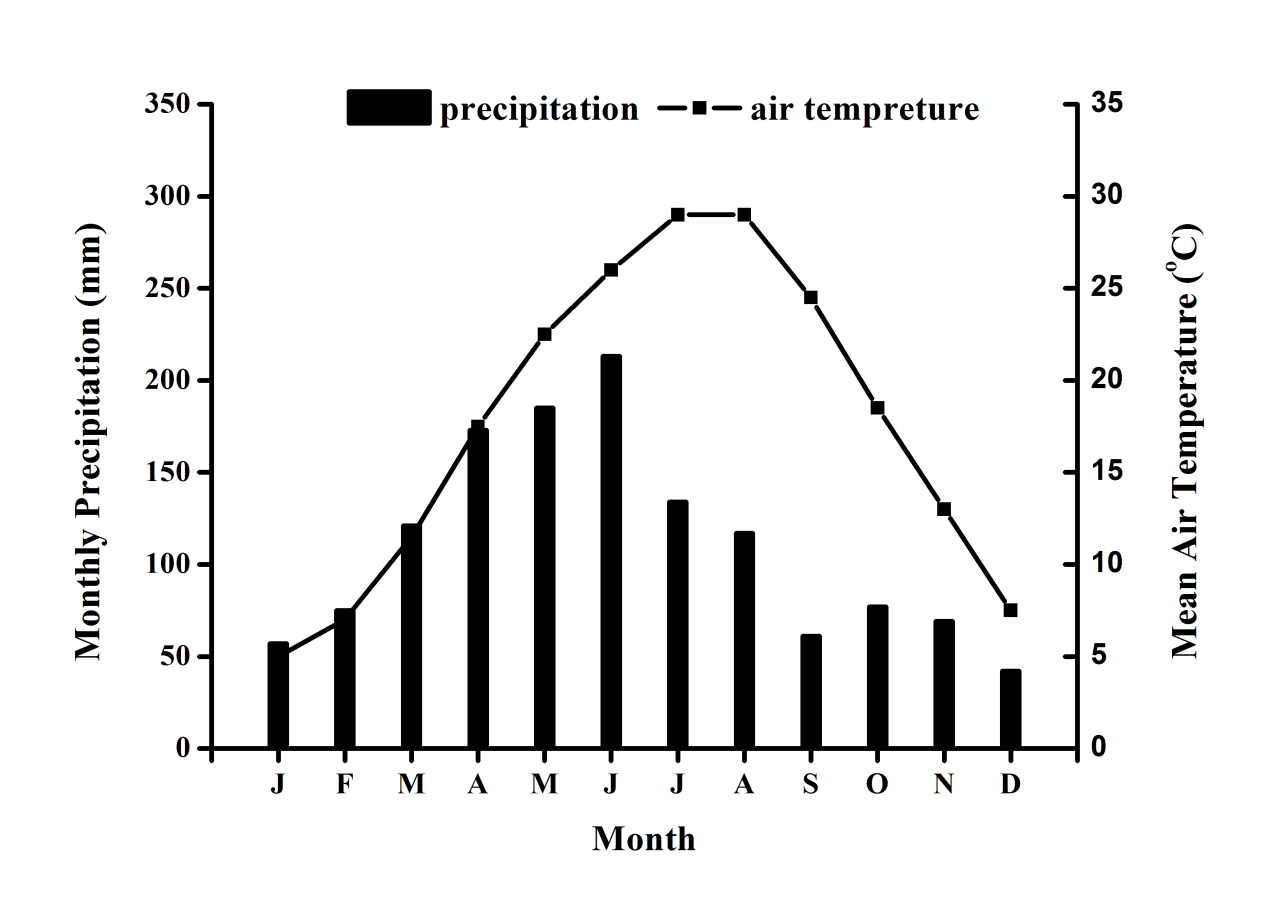


**Fig. S1.** Total monthly precipitation and mean monthly air temperature (2014)
